# The Urgent Need for Dengue Vaccination: Combating an Escalating Public Health Crisis in Pakistan

**DOI:** 10.3390/vaccines12080913

**Published:** 2024-08-13

**Authors:** Somia Iqtadar, Javed Akram, Amjad Khan

**Affiliations:** 1 Dengue Expert Advisory Group, Lahore 54000, Pakistan; somia.iqtadar@gmail.com; 2 Department of Medicine, King Edward Medical University, Lahore 54000, Pakistan; 3 Pakistan Society of Internal Medicine (PSIM), Lahore 54000, Pakistan; jakramaimc@gmail.com; 4 Asia Dengue Voice & Action Next Generation (ADVA NexGen) Group, 8 Fleming Road, Wanchai, Hong Kong, China; 5 Department of Oncology, University of Oxford, Oxford OX3 7DQ, UK

**Keywords:** dengue fever, dengue vaccination, Pakistan, tropical diseases

## Abstract

Dengue fever, caused by the dengue virus (DENV), poses a significant global health threat, with a dramatic increase in cases driven by climate change, urbanization, and mosquito resistance. In Pakistan, a country with a population of 240 million, the world’s fifth largest, dengue has emerged as an escalating public health crisis, with seasonal outbreaks severely straining the healthcare system. Despite decades of vector control efforts, there has not been much success, necessitating the introduction of dengue vaccination to boost population immunity. Recent advancements in vaccine development demonstrate promising efficacy and safety profiles, even in dengue-naive individuals. Implementing a dengue vaccination program in Pakistan could significantly reduce the disease burden, lower healthcare costs, and prevent future outbreaks. Integrating vaccination with existing public health initiatives can achieve high coverage and improve overall public health outcomes.

## 1. Introduction

Dengue fever, also known as “breakbone fever”, caused by the dengue virus (DENV), is the fastest-growing tropical disease and a significant global public health threat. Each year, there are an estimated 390 million DENV infections, with 96 million of these cases manifesting clinically [1]. As of 30 April 2024, more than 7.6 million dengue cases have been reported to the World Health Organization (WHO) in 2024, with 3.4 million confirmed cases, over 16,000 severe cases, and more than 3000 deaths [2]. It is estimated that around 3.9 billion people in 128 countries are at increased risk of dengue infection [3]. This rapid expansion is driven by several factors, including climate change, deforestation associated with uncontrolled urbanization, overpopulation, and the emergence of mosquitoes resistant to common insecticides. DENV infection results in a spectrum of clinical manifestations, ranging from asymptomatic to self-limiting dengue fever (DF) to severe and potentially fatal illnesses such as dengue hemorrhagic fever (DHF) and dengue shock syndrome (DSS). DHF and DSS pose significant threats to human health, particularly in tropical and subtropical regions. There is no known specific antiviral treatment for dengue/severe dengue, and the management is symptomatic and supportive (antipyretics, judicious intravascular volume repletion).

**Figure 1:**
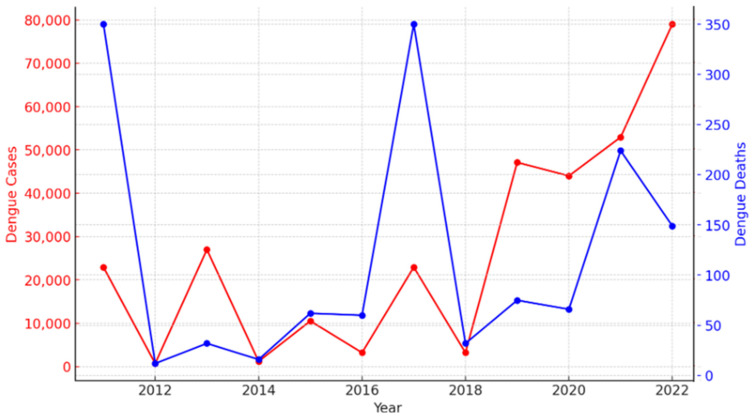
Dengue cases and deaths in Pakistan during the various outbreaks between 2011 and 2022, highlighting the urgent need for a dengue vaccination program to control outbreaks and prevent the loss of further lives [6,7,8,9].

Asia bears 70% of the global dengue burden, with Pakistan, the world’s fifth largest population of 240 million, among the endemic countries experiencing seasonal surges in cases and the circulation of different dengue serotypes (DENV-1, DENV-2, DENV-3, DENV-4). In 2011, a devastating first large dengue outbreak of 22,938 cases was reported in Lahore, the capital city of the Punjab, in which at least 350 people died due to DHF and DSS [4]. Since 2011, Pakistan has experienced periodic and increasingly frequent outbreaks of dengue, which have placed a significant burden on the country’s healthcare system (Figure 1) [5]. Over the past decade, dengue cases have surged dramatically, with a notable increase in both morbidity and mortality rates. The year 2019 saw one of the highest outbreaks with 47,120 cases and 75 deaths, [6] while 2022 alone reported 79,007 confirmed dengue cases, including 149 deaths [7]. 

This significant burden of these recurring dengue outbreaks places immense strain on healthcare facilities in Pakistan in many ways. These include the following:

**1. Overcrowded Hospitals:** The high number of dengue cases leads to overcrowding in hospitals, which results in a shortage of beds and essential medical supplies. This situation can compromise the quality of care not only for dengue patients but also for those suffering from other ailments.

**2. Healthcare Workforce Stress:** The surge in dengue cases demands increased attention and resources from healthcare professionals, leading to burnout and overwork. The diversion of medical staff to handle dengue patients can also detract from their ability to manage other critical health issues.

**3. Economic Impact:** The cost of medical care for dengue patients, including hospitalization, medication, and supportive care, places a substantial financial burden on both the healthcare system and the affected families. This economic strain is exacerbated by lost productivity due to illness and caretaking responsibilities. 

**4. Public Health Resources:** Managing dengue outbreaks requires significant public health resources, including vector control measures, public awareness campaigns, and monitoring and surveillance systems. The allocation of these resources to combat dengue can detract from other public health initiatives. 

**5. Morbidity and Mortality:** The morbidity associated with dengue fever, including severe cases that can lead to DHF or DSS, increases the risk of hospitalization, worsens comorbidity, and increases the chances of mortality. The fatalities resulting from severe dengue cases add to the human cost of the epidemic. 

## 2. Need for Dengue Vaccination in Pakistan 

Despite decades of comprehensive vector control efforts in Pakistan, success in curbing dengue has been limited. Dengue control programs at both the national and regional levels have focused on strategies to reduce dengue by decreasing the population of Aedes mosquitoes, the vectors that transmit dengue viruses [6,7]. Additionally, the presence of *Aedes albopictus* in Afghanistan and Iran contributes to the spread of dengue in the region, and substantial population movement between Pakistan and these bordering neighboring countries, particularly Afghanistan and Iran, affects the dynamics of dengue transmission [6]. With changing disease dynamics, the spread of the virus through global travel, climate change, and rapid urbanization favoring mosquito breeding, the traditional reliance on vector control alone, as advocated by the WHO, is insufficient to prevent dengue outbreaks in Pakistan. This inadequacy is evidenced by periodic and increasingly frequent dengue outbreaks in Pakistan since 2011.

To complement vector control efforts, there is a critical need for dengue vaccines in Pakistan to boost overall population immunity. A parallel can be drawn to the COVID-19 pandemic, where measures such as masks and lockdowns were only partially effective [10,11]; it ultimately took vaccines to bring the pandemic under control. Similarly, the introduction of a dengue vaccination program is essential to reduce the incidence and severity of dengue infections, thereby alleviating the burden on the healthcare system and improving public health outcomes.

## 3. Dengue Vaccine Development

The four distinct types of the dengue virus (DENV-1, 2, 3, and 4) exhibit significant genetic differences, resulting in immunity conferred by infection with one type being specific only to that particular serotype, without providing protection against the others. Consequently, individuals can remain susceptible to multiple dengue infections over time. Secondary heterologous dengue, where a person contracts a different serotype following a previous infection, poses a well-documented risk for antibody-dependent enhancement (ADE), which can lead to severe disease [12]. However, third and fourth infections typically result in milder or even asymptomatic cases due to the broader immune response generated by exposure to two different dengue serotypes, offering a degree of cross-protection. As a consequence of the aforementioned factors, dengue vaccines typically exhibit lower efficacy in individuals who have never experienced a previous dengue infection (dengue-naive) compared to those who have had at least one prior infection. 

In theory, an ideal vaccine should elicit a balanced, long-lasting immunity against all four DENV serotypes. However, in practical terms, this may not be strictly necessary, as the immune responses triggered by two distinct dengue infections are often broad enough to confer adequate protection. Therefore, an acceptable vaccine should be capable of eliciting immune responses similar to those induced by at least two separate natural dengue infections. Such a vaccine-induced immune response, akin to that induced by experiencing two dengue infections, should theoretically safeguard vaccinated individuals from developing severe disease, even among those who are dengue-naive. 

Vaccination has been widely acknowledged as a crucial component of a comprehensive strategy to mitigate the global impact of dengue. For over 75 years, scientists and developers have strived to design and improve dengue vaccine candidates, but they have faced significant and formidable obstacles [13,14]. The first dengue vaccine, Dengvaxia^®^ (CYD-TDV), developed by Sanofi Pasteur, Lyon, France, [15] became available in 2015 and has been approved in over 20 countries, including the US, the EU, some Asian countries, including Indonesia and the Philippines, and some Latin American countries, including Mexico, Brazil, El Salvador, Costa Rica, Paraguay, and Peru. Dengvaxia^®^ aims to work by stimulating the immune system to produce antibodies against all four dengue serotypes, DENV-1, DENV-2, DENV-3, and DENV-4, thereby reducing the risk of severe dengue illness upon subsequent exposure to the virus. It is recommended for individuals aged 9–45 years old who have had a prior dengue infection, as its efficacy is higher in this group, reducing severe dengue cases by about 80%. However, due to its design, Dengvaxia^®^ does not elicit the same range of immune responses as those generated by two distinct dengue infections. Consequently, despite its efficacy in dengue-experienced individuals, longer-term safety data demonstrated that this vaccine increased the risk of more severe symptoms upon DENV infection in individuals who were dengue-naive and experienced breakthrough infections (infections that occur despite vaccination) and children younger than 9 years [15,16,17]. As a result, the recommendation for Dengvaxia^®^’s use was restricted to individuals who have previously been infected with dengue. This limitation has hindered the widespread deployment of this vaccine. In 2019, the U.S. Food and Drug Administration (FDA) approved Dengvaxia^®^ for use in endemic regions of the United States, specifically for individuals aged 9–16 years old who have previously had a laboratory-confirmed dengue infection. The WHO recommends Dengvaxia^®^ for individuals in endemic areas with a confirmed previous dengue infection to maximize the benefits and minimize the risks [18]. 

Another vaccine, Qdenga^®^ (TAK-003) by Takeda, has shown promising results, with an overall efficacy of approximately 80% in preventing symptomatic dengue in children and adolescents in endemic countries [19,20,21,22]. TAK-003 has been licensed for the prevention of dengue in the European Union, the UK, Brazil, Argentina, Thailand, and Indonesia, among other countries, for individuals aged 4–60 years old regardless of prior dengue exposure, providing a significant advantage in broader population protection. In clinical trials and long-term follow-up studies, Qdenga^®^ demonstrated efficacy in protecting against infection with both DENV-1 and DENV-2, irrespective of whether individuals had prior exposure to dengue (seropositive) or were dengue-naive. However, it was found to only provide protection against DENV-3 in individuals with previous dengue infection but not in dengue-naive individuals. Data from the clinical trials were insufficient to conclusively determine whether the vaccine offers protection against DENV-4 in dengue-naive individuals, as there were too few cases to draw firm conclusions. A notable difference between Qdenga^®^ and Dengvaxia^®^ lies in the absence of evidence indicating an increased risk of severe dengue among dengue-naive recipients of Qdenga^®^. Consequently, Qdenga^®^ has been authorized for use, even in individuals without prior dengue exposure. In May 2024, the WHO officially recommended TAK-003 for children aged 6–16 years old in regions with a high dengue burden and in which transmission presents a significant public health challenge [23]. 

More recently, a third vaccine, known as TV003, developed by the U.S. National Institutes of Health (NIH) and produced by Instituto Butantan in Brazil/Merck, has shown promising results in clinical trials, with an efficacy rate of about 80% [24,25,26,27,28]. TV003 is designed to provide protection against all four dengue virus serotypes and has been tested in various age groups, demonstrating a favorable safety and efficacy profile. The trial findings indicate robust immune responses against all four dengue serotypes, with protection observed against both DENV-1 and DENV-2, even among individuals with no prior dengue exposure. However, its effectiveness against DENV-3 and DENV-4 remains undetermined due to the insufficient number of cases in the trial. Further results from longer-term follow-ups are eagerly anticipated. TV003 is currently in the advanced stages of development and has the potential to be another effective tool in the fight against dengue. 

Both Qdenga^®^ and TV003 have a notable advantage over Dengvaxia^®^ in their ability to induce “killer” T cells against all four dengue viruses. This crucial aspect is absent in Dengvaxia^®^. Killer T cells play a vital role in eliminating virus-infected cells from the body, thereby preventing severe disease [29,30,31]. Consequently, dengue-naive individuals who receive either Qdenga^®^ or TV003 are shielded from severe disease, even in cases of breakthrough infection. Importantly, they do not face an elevated risk of severe disease. 

## 4. Wait or Waste

Drawing parallels from the COVID-19 vaccination experience, we can devise strategies for the implementation of dengue vaccination in Pakistan. Despite not being perfect, COVID-19 vaccines played a crucial role in reducing severe cases, hospitalization rates, and mortality, ultimately aiding in controlling the pandemic. While none of the dengue vaccines currently available, either licensed or in the developmental phase, can be considered flawless, we strongly believe that they represent significant advancements. Drawing upon our collective understanding of dengue vaccine science, we believe these vaccines are sufficiently effective and safe to complement vector control efforts, thereby reducing the overall burden of dengue.

To complement vector control efforts, introducing dengue vaccines is imperative to enhancing the overall population immunity in Pakistan. Dengue is a growing concern in Pakistan, with frequent outbreaks causing significant morbidity and mortality, straining the healthcare system, and affecting the economy due to loss of productivity. The country has reported thousands of dengue cases in recent years, with many resulting in severe disease and fatalities. The implementation of a dengue vaccination, particularly Qdenga^®^ due to its broader age approval and efficacy without the requirement for prior infection, could substantially reduce the burden of the disease, lower healthcare costs, and prevent outbreaks. Concurrently, enhancing vector control measures such as intensifying larval source reduction, deploying advanced surveillance technologies like Geographic Information System (GIS) mapping, and fostering community participation in mosquito breeding site elimination could synergistically bolster these vaccination efforts. Public health interventions, combining widespread vaccination with rigorous vector control, can lead to herd immunity, significantly reducing the transmission rates and protecting vulnerable populations such as children, the elderly, and immuno-compromised individuals. The existing vaccination policy in Pakistan, guided by the Ministry of National Health Services, Regulations, and Coordination (NHSRC), ensures comprehensive coverage of routine vaccines through the National Immunization Program (NIP). This established infrastructure and capacity can facilitate the integration of dengue vaccination, ensuring high coverage and efficient resource utilization. Moreover, integrating dengue vaccination with existing immunization programs in line with the WHO recommendations could significantly enhance dengue prevention efforts in the country, ultimately improving public health outcomes.

## Data Availability

The data presented in this study is available in this article.
